# The Lipopolysaccharide-Sensing Caspase(s)-4/11 Are Activated in Cirrhosis and Are Causally Associated With Progression to Multi-Organ Injury

**DOI:** 10.3389/fcell.2021.668459

**Published:** 2021-07-15

**Authors:** Ugo Soffientini, Nigel Beaton, Sukriti Baweja, Emmanuel Weiss, Chhagan Bihari, Abeba Habtesion, Vishal Patel, Valerie Paradis, Archana Sharma, Tu Vinh Luong, Andrew Hall, Aida Nadar, Shiv Sarin, Shilpa Chokshi, Roger Williams, Benedicte Py, Richard Moreau, Rajiv Jalan, Gautam Mehta

**Affiliations:** ^1^Institute for Liver and Digestive Health, UCL, London, United Kingdom; ^2^Institute of Hepatology, Foundation for Liver Research, London, United Kingdom; ^3^Institute of Liver and Biliary Sciences, New Delhi, India; ^4^Département d’Anesthésie-Réanimation, Hôpital Beaujon, Assistance Publique-Hôpitaux de Paris, Clichy, France; ^5^UMR S1149, Inserm, University of Paris, Paris, France; ^6^School of Immunology and Microbial Sciences, King’s College London, London, United Kingdom; ^7^Institute of Liver Studies, King’s College Hospital NHS Foundation Trust, London, United Kingdom; ^8^Département d’Hépatologie, Hôpital Beaujon, Assistance Publique-Hôpitaux de Paris, Clichy, France; ^9^INSERM U1111, CNRS UMR 5308, Centre International de Recherche en Infectiologie (CIRI), ENS de Lyon, Université Claude Bernard Lyon 1, Lyon, France

**Keywords:** cirrhosis, lipopolysaccharide, caspase, liver failure, endotoxin, dysbiosis, pyroptosis

## Abstract

**Background and Aims:**

The development of multi-organ injury in cirrhosis is associated with increased intestinal permeability, translocation of gut-derived bacterial products [e.g., lipopolysaccharide (LPS)] into the circulation, and increased non-apoptotic hepatocyte cell death. Pyroptosis is a non-apoptotic, lytic form of cell death mediated by the LPS-sensing caspase(s)-4/11 (caspase-4 in humans, caspase-11 in mice), which leads to activation of the effector protein Gasdermin D (GSDMD) and subsequent formation of pores in the plasma membrane. Endoplasmic reticulum (ER) stress, a feature of cirrhosis, has been identified as a factor promoting the activation of caspase-11, thus increasing sensitivity of the cell to LPS-mediated pyroptosis. The aim of this study was to determine the role of bacterial LPS in the activation of hepatic caspase(s)-4/11 and progression of hepatic and extra-hepatic organ injury in cirrhosis.

**Materials and Methods:**

Human liver samples from patients with stable cirrhosis (SC) or acutely decompensated cirrhosis (AD) were analyzed for caspase-4 activation by immunohistochemistry. Wild-type and *Casp11*^–/–^ mice underwent CCl_4_ treatment by gavage to induce advanced liver fibrosis, and subsequently low-dose injection of LPS to mimic bacterial translocation and induce multi-organ injury. Liver, kidney, and brain function were assessed by plasma ALT/creatinine and brain water respectively. The activity of inflammatory caspases was assessed by fluorometric assay and the occurrence of pyroptosis and overall cell death in liver tissue by GSDMD cleavage and TUNEL assay, respectively. Primary human hepatocytes were cultured according to standard techniques.

**Results:**

Human liver samples demonstrated increased caspase-4 activation in AD cirrhosis. Caspase-4 activation was associated with MELD score and circulating levels of LDH. Wild-type mice treated with CCl_4_ developed significant multi-organ injury (increased ALT, creatinine, and brain water) upon LPS injection, and showed increased hepatic GSDMD cleavage compared to mice treated with CCl_4_ alone. Primary human hepatocytes could be sensitized to pyroptosis by pre-treatment with the ER-stress inducer tunicamycin and LPS. *Casp11*^–/–^ mice treated with CCl_4_ + LPS were significantly protected from multi-organ injury compared to wild-type CCl_4_ + LPS.

**Conclusion:**

These data demonstrate for the first time a causal relationship between LPS-mediated activation of caspase(s)-4/11 and development of hepatic and extra-hepatic injury in cirrhosis.

## Graphical Abstract

Model of caspase-4/11 activation in cirrhosis. Gut-derived bacterial LPS (1) enters the portal circulation after translocation across the gut epithelium and is internalized by hepatocytes. Cytoplasmic LPS is recognized by caspase-4/11 (2), which undergoes self-activation upon ligand binding. The activity of caspase-4/11 is enhanced by endoplasmic reticulum stress (3), which occurs in fibrosis/cirrhosis, leading to “sensitization” of this pathway. Active caspase-4/11 cleaves the dimeric protein Gasdermin D (GSDMD) (4), and freeing the N-terminal domain to migrate to the plasma membrane and form GSDMD N-terminal octameric pores (5). GSDMD pores insert themselves into the plasma membrane, allowing the deregulated passage of molecules and causing cell swelling and membrane rupture, eventually resulting in pyroptotic cell death (6).

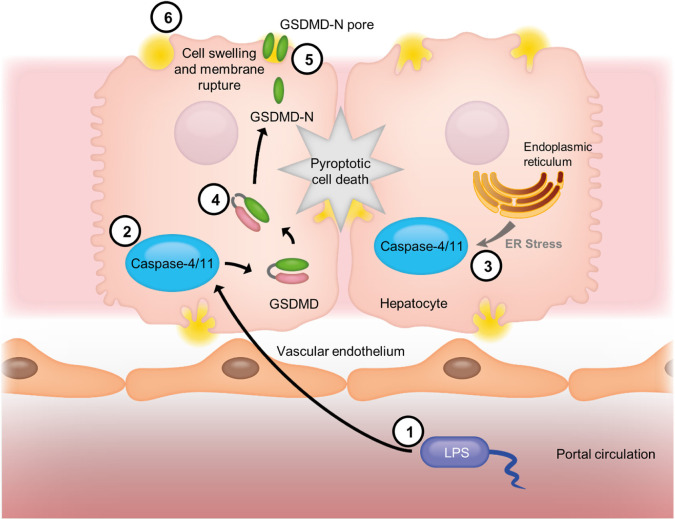

## Introduction

Cirrhosis is responsible for over 1 million global deaths each year, equating to 2% of deaths worldwide (Mokdad et al., 2014). The natural history of cirrhosis is considered to be a progression through an asymptomatic compensated phase, to a decompensated state following the development of acute complications such as jaundice, variceal bleeding, ascites or hepatic encephalopathy (HE). Amongst patients with acutely decompensated cirrhosis (AD), a subgroup of 1 in 3 patients develop severe hepatic and extra-hepatic organ injury – a condition that has been termed acute-on-chronic liver failure (ACLF), and is associated with high short-term mortality (Moreau et al., 2013).

The mechanisms determining susceptibility to multi-organ injury in cirrhosis remain undetermined, but clear associations have been shown with systemic inflammation and altered host response to injury (Clària et al., 2016). The most common precipitants of multi-organ injury in cirrhosis are bacterial infection and alcoholic hepatitis (Moreau et al., 2013). In patients without an identified precipitating event, it is considered likely that translocation of bacterial products from the intestinal lumen contributes to systemic inflammation and multi-organ dysfunction (Markwick et al., 2015). Indeed, the presence of circulating bacterial products is a predictor of multi-organ injury and prognosis in cirrhosis (Michelena et al., 2015).

The major consequence of bacterial translocation (BT) in cirrhosis is systemic inflammation, through the action of pathogen-associated molecular patterns (PAMPs) such as bacterial lipopolysaccharide (LPS). Our group, and others, have shown circulating levels of LPS to be elevated in patients with severe alcoholic hepatitis, and to be a predictor of multiple organ failure and mortality (Markwick et al., 2015; Michelena et al., 2015). Additionally, increased host sensitivity to bacterial PAMPs has been noted in cirrhosis, with exaggerated *ex vivo* pro-inflammatory cytokine responses to LPS in humans and animal models (Tazi et al., 2007; Gandoura et al., 2013).

In parallel, there is accumulating evidence to support a role for excessive cell death in development of multi-organ failure in cirrhosis. We have previously shown that acute decompensation in cirrhosis is associated with elevated circulating markers of cell death, which progressively increase with disease severity (Macdonald et al., 2018). Moreover, the mode of cell death also changes with disease severity, from predominantly apoptotic to non-apoptotic cell death with development of multi-organ dysfunction. This may partly explain the negative clinical results from efforts to inhibit apoptosis in acute decompensation of cirrhosis using pan-caspase inhibitors (Mehta et al., 2018).

A direct link between LPS-sensing and non-apoptotic cell death has recently been described. Pyroptosis is a non-apoptotic, lytic mode of cell death mediated through inflammatory caspases, also termed the “non-canonical inflammasome.” Intracellular LPS is directly sensed by caspase-4 (human)/caspase-11 (mouse) which directly leads to activation of this pathway through cleavage of the cytoplasmic protein Gasdermin-D (GSDMD). Subsequently, the N-terminal fragment of GSDMD forms pores in the plasma membrane which may precipitate lytic cell death, release of damage-associated molecular patterns (DAMPs), and amplification of local inflammatory responses (Ding et al., 2016). Unlike apoptosis, which is immunologically silent, pyroptosis is an immunogenic form of cell death. This is of potential benefit to the host in the elimination of intracellular pathogens. However, in the context of cirrhosis, high levels of hepatocyte pyroptosis in response to circulating LPS may lead to greater liver dysfunction, increased systemic inflammation and multi-organ failure. A link between endoplasmic reticulum stress, a feature of cirrhosis, and activation of LPS-sensing caspases has also been described, providing a potential mechanism for sensitization of pyroptosis pathways in cirrhosis (Endo et al., 2006).

The aim of the present study was to evaluate the role of LPS-sensing caspase(s)-4/11 in hepatic and extra-hepatic organ injury in cirrhosis. Cirrhosis is associated gut bacterial dysbiosis, the degree of which correlates with the severity of the disease (Bajaj, 2019). Moreover, cirrhosis is also associated with increased intestinal permeability, and it has been recently demonstrated that translocation of bacterial products to the circulation underpins the sensitization of hepatocytes to cell death that is observed in chronic liver disease (Isaacs-Ten et al., 2020). The hypothesis of this study was that activation of caspase(s)-4/11 and high levels of hepatic pyroptosis are causally related to the development of multi-organ injury in cirrhosis. To interrogate this hypothesis, we characterized activation of caspase-4 in liver tissue from patients with stable compensated and acutely decompensated cirrhosis, and explored the activation of caspase-11 and GSDMD in a mouse model of liver fibrosis with multi-organ injury. Further, we used *Casp11*^–/–^ mice to dissect the role of this pathway in hepatic and extra-hepatic organ injury.

## Materials and Methods

### Human Liver Samples

Liver biopsy specimens formalin-fixed and paraffin-embedded (FFPE) were available from the Institute for Liver and Biliary Sciences (ILBS) Biobank, from an established cohort of 79 hospitalized patients presenting with acutely decompensated (AD) alcohol-related cirrhosis, and a further 10 outpatients with stable, compensated (SC) alcohol-related cirrhosis. Clinical data from these patients was collected from the time of biopsy, and Child-Pugh (CP) and Model for End Stage Liver Disease (MELD) scores calculated. Ethical approval was granted by the ILBS IRB (Ref: IEC/2019/71/MA07, November 02, 2019).

### Mouse Model of Advanced Fibrosis and Multi-Organ Dysfunction

Male C57BL/6J mice, wild type (Charles Rivers, United Kingdom) or Casp11^–/–^, were used for all experiments. Casp11^–/–^ mice have a targeted deletion of 16 amino acids from exon 5 of *casp-11*, including the QACRG enzymatic active site (Wang et al., 1998). All mice were housed in a temperature and light controlled (12 hours light/dark cycle) facility at the Comparative Biology Unit, UCL, and received standard chow and water *ad libitum*. All procedures were performed in accordance with United Kingdom Home Office Animals (Scientific Procedures) Act 1986 (updated 2012). The study was approved by the University College London Animal Welfare and Ethical Review Board (AWERB) and conducted with a United Kingdom Home Office project license.

The model of advanced fibrosis and multi-organ dysfunction was previously described by Sanyal and colleagues (Carl et al., 2016). Briefly, advanced fibrosis was induced by gavage of carbon tetrachloride (CCl_4_ 0.5ml/kg, 1:1 olive oil, 20 doses over 10 weeks). Control mice were treated with olive oil alone. Subsequently, low-dose *Klebsiella pneumoniae* lipopolysaccharide (LPS) (Merck, United Kingdom) was injected intraperitoneally (i.p) at 2–4 mg/kg to mimic bacterial translocation and induce ACLF, or equivalent volume of 0.9% saline as control. For some experiments, a high-dose of LPS (12 mg/kg) was injected in naïve mice. All experiments were terminated at 4 h following intervention.

### Histopathological Assessment and Immunostaining

Human and mouse samples were formalin fixed, paraffin embedded and sections cut according to standard techniques. Human samples underwent immunostaining for Caspase-4 (Caspase-4 polyclonal antibody raised against AA: 95–137, AMS Bio, United Kingdom) according to manufacturer’s protocols. Slides were reviewed by two investigators (CB and AS) blind to clinical characteristics. Under high magnification, 20 consecutive high-power fields (hpfs) were selected and the positive staining cells (dark brown cytoplasmic staining) were counted. The mean scores of both investigators were taken, and data expressed as average positive cells/hpf. Mouse sections were stained with hematoxylin and eosin (H&E) or picro-Sirius red, and collagen proportionate area (CPA) calculated as previously described (Hall et al., 2013). Additionally, terminal deoxynucleotidyl transferase dUTP nick end labeling (TUNEL) staining was performed on mouse liver sections (*In Situ* Cell Death Detection Kit, POD – Roche Diagnostics, United Kingdom) according to manufacturer’s protocols. Degree of cell death was quantified by analysis of immunohistochemical positive areas measured by FIJI Image J software as described previously (Hall et al., 2013).

### Characterization of Organ Dysfunction in Mouse Models

Mouse plasma alanine aminotransferase (ALT) and creatinine concentration were measured by Cobas Integra 400 automated analyzer (Roche Diagnostics, Burgess Hill, United Kingdom) using the relevant kits according to the manufacturer’s instructions. Plasma lactate dehydrogenase (LDH) was also measured as a circulating marker of non-apoptotic cell death, using the LDH-Glo Cytotoxicity Assay (Promega, United Kingdom). Brain tissue water content was measured according to a previously described gravimetric technique (Marmarou et al., 1982). Circulating levels of LPS were measured by end-point chromogenic endotoxin detection assay based on the amebocyte lysate method (Thermo Fisher Scientific, United Kingdom).

### Cell Culture

Cryopreserved primary human hepatocytes (Lonza Biologics, United Kingdom) were cultured with HCM Thawing Medium, Hepatocyte Plating Medium and HCM Hepatocyte Culture Medium (Lonza Biologics, United Kingdom) according to supplier’s instructions. For some experiments cells were exposed to LPS from *Klebsiella pneumoniae* (Merck, United Kingdom) or tunicamycin (Merck, United Kingdom) according to the doses stated.

### Protein Expression Analysis

Proteins were isolated from snap frozen human and mouse tissue samples and cell culture samples, by standard techniques and analyzed by Western blot. In brief, frozen tissues were aliquoted (50–100 mg) into screw cap tube (Starlab, United Kingdom) containing 1mm glass beads (Merck, United Kingdom) and homogenized in PBS using a Precellys 24 Tissue Homogenizer (Bertin Instruments, France). 2× RIPA buffer (Merck, United Kingdom) and protease inhibitor cocktail (Roche Diagnostics, Burgess Hill, United Kingdom) was added to the homogenate and incubated at 4°C with agitation for 10 min. Tubes were then centrifuged at 15.000*g* × 15 min and the supernatant collected aliquoted and stored at −80°C for future analysis. Cell culture samples were processed on-plate. Briefly, culture medium was removed and 20 μl/cm^2^ RIPA buffer (protease inhibitor added to each well or flask. Cells were detached with a rubber policeman (Thermo Fisher scientific, United Kingdom), transferred to microcentrifuge tubes, incubated at 4°C with agitation for 10 min, then centrifuged at 15.000*g* × 15 min. Supernatant were collected, aliquoted, and stored at −80°C for future analysis. Blots were probed using the primary antibodies described in Supplementary Table 1A. Immune complexes were detected using horseradish peroxidase (HRP)-conjugated secondary antibodies (Cell Signaling Technology, United States) and enhanced chemiluminescence (ECL) reagents (BioRad, United Kingdom). Densitometric quantification was performed using ChemiDoc imaging stem and Image Lab software (BioRad, United Kingdom).

### Messenger RNA Expression Analysis

Total RNA was extracted from snap frozen mouse liver and cell samples using TRI reagent (Merck, United Kingdom), and retrotranscribed using AffinityScript cDNA synthesis kit (Agilent, United Kingdom). Subsequently, gene expression was analyzed according to manufacturer’s protocols, using the primers described in Supplementary Table 1B.

### Measurement of Caspase Activity

Caspase-1 and Caspase-11 activity was measured in mouse liver homogenate using a fluorometric assay (Abcam, United Kingdom) as previously described (Khanova et al., 2018). Caspase-1 activity was measured by cleavage of the motif WEHD, and Caspase-11 by cleavage of the motif LEVD.

### Statistics

Variables are presented as mean ± standard error, or median and interquartile range, depending on normal or non-normal distribution. Data were analyzed by *t*-test (with Welch correction where necessary), Mann-Whitney test, one-way ANOVA (with Tukey’s *post hoc* test), Kruskal-Wallis (with Dunn’s *post hoc* test), Pearson’s or Spearman correlation as appropriate, using GraphPad Prism (version 5.03 for Windows; GraphPad Software, San Diego, CA, United States) and Minitab17 (Minitab, Inc. State College, PA, United States).

## Results

### Caspase-4 Expression Is Increased in Liver Tissue of Patients With Acutely Decompensated Cirrhosis and Correlates With Disease Severity

**FIGURE 1:**
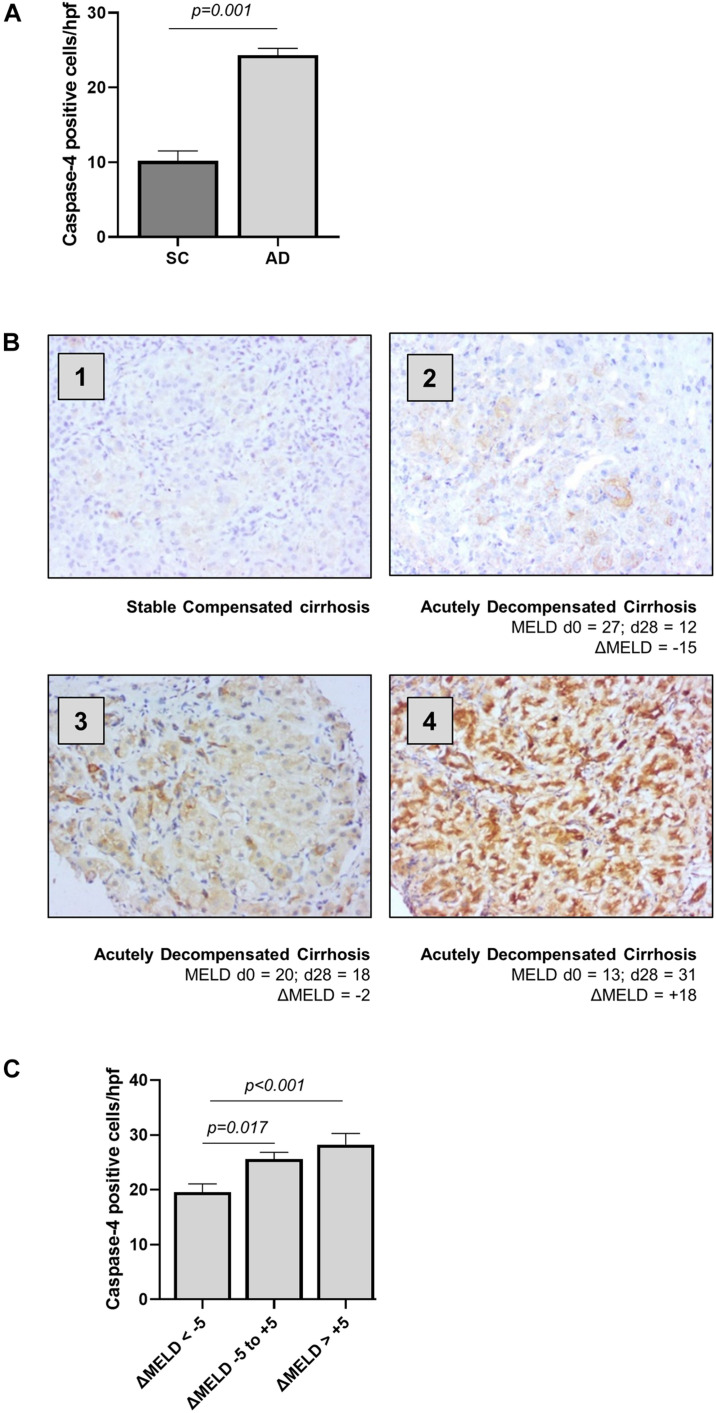
Hepatic caspase-4 expression correlates with the severity of liver disease. The expression of caspase-4 in the liver was measured by immunohistochemistry and quantified as number of positive cells/high power field. **(A)** Patients with acutely decompensated cirrhosis were found to have elevated expression of caspase-4 in the liver compared to patients with stable, compensated cirrhosis (Mann-Whitney *U* test, *p* < 0.001). **(B)** Patients were divided into three categories, based on ΔMELD score (MELD d28 – MELD d0): ‘Improved’ patients with ΔMELD below –5, “stable” with ΔMELD between –5 and + 5, and “worsened” with an increase in MELD score greater than + 5 points. Panels 1–4 show representative caspase-4 staining for (1) stable cirrhosis (SC), (2) ‘improved’ AD, (3) “stable” AD, and (4) “worsened” AD. **(C)** Caspase-4 expression in the liver was significantly different among the three groups (Kruskal-Wallis test *p* < 0.001), being significantly higher in “stable” and “worsened” groups, compared to ‘improved’ patients (Dunn’s test, *p* = 0.017 and *p* < 0.001 respectively).

Clinical characteristics for outpatients with stable compensated cirrhosis (SC) and hospitalized patients with acutely decompensated cirrhosis (AD) are presented in Table 1. The expression of caspase-4 in the liver was measured by immunohistochemistry, employing an antibody raised against the central portion of caspase-4 (AA range: 95–137) and quantified as number of positively stained cells per high power field. Abundance of caspase-4 in hepatocytes was significantly increased in AD patients, compared to stable cirrhotic controls (Figure 1A), and circulating LDH levels, a marker of non-apoptotic cell death, were significantly correlated with hepatic expression of caspase-4 in AD (*r* = 0.3287, *p* = 0.026). In AD, the expression of caspase-4 was also significantly correlated with disease severity by MELD score at time of biopsy (*r* = 0.2700, *p* = 0.011). By contrast, no significant correlation of caspase-4 expression with MELD score was noted in stable compensated patients (*r* = 0.01972, *p* = 0.666). In hospitalized patients with AD cirrhosis, further laboratory tests were available at 14-days and 28-days following liver biopsy; hepatic caspase-4 expression was also significantly correlated with MELD score at day 14 post-biopsy (*r* = 0.2587, *p* = 0.027), and more strongly correlated with MELD score at day 28 post-biopsy (*r* = 0.4800, *p* < 0.001). To explore the influence of hepatic caspase-4 expression on disease trajectory, we examined change in MELD score at day 28 (delta MELD) in AD cirrhosis patients. In patients with decompensated cirrhosis, a change in MELD score of 5 points at 1 month has been shown to predict short-term mortality (Merion et al., 2003). Accordingly, AD patients were classified into three groups based on delta MELD [improved (< –5; *n* = 22), stable (–5 to + 5; *n* = 30), and worsened (> + 5; *n* = 25)]. The level of caspase-4 expression across the three groups was significantly different by multivariate analysis (Figures 1B,C), with *post hoc* testing showing a significant increase in caspase-4 expression between the ‘improved’ group and both the “stable” and “worsened” (improved vs. stable: 20.02 vs. 26.17, *p* = 0.017; improved vs. worsened: 20.02 vs. 30.48; *p* < 0.001). Taken together, these data demonstrate an association of hepatic caspase-4 expression with severity of liver disease in cirrhosis, and a further association with disease trajectory in hospitalized patients with decompensated cirrhosis.

**TABLE 1:** Patient characteristics for liver tissue characterization.

	Stable compensated cirrhosis (SC) *n* = 10	Acutely decompensated cirrhosis (AD) *n* = 79
Age [mean (SEM)]	44.9 (3.2)	45.5 (1.0)
Male [n (%)]	10 (100)	78 (99)
***Cause of acute decompensation [n (%)]***		
Infection	N/A	15 (18.9)
Alcohol	N/A	26 (67.0)
GI bleeding	N/A	4 (5.0)
Unknown	N/A	7 (8.8)
***Aetiology [n (%)]***		
Alcohol	10 (100)	79 (100)
***Clinical data at time of biopsy***		
Bilirubin [median (IQR)]	0.75 (0.60–0.95)	20.2 (13.45–28.37)
INR [median (IQR)]	1.00 (0.93–1.00)	1.99 (1.75–2.29)
Creatinine [median (IQR)]	0.60 (0.50–0.70)	0.54 (0.34–0.78)
MELD score [median (IQR)]	6.00 (6.00–6.75)	19.66 (16.08–23.79)
***Child-Pugh Class***		
A	10 (100)	18 (23)
B	0 (0)	59 (75)
C	0 (0)	2 (2.4)
***Clinical data at 28 days***		
Bilirubin [median (IQR)]	N/A	10.6 (5.77–20.70)
INR [median (IQR)]	N/A	1.62 (1.14–2.00)
Creatinine [median (IQR)]	N/A	1.00 (0.59–1.50)
MELD score [median (IQR)]	N/A	19.16 (13.31–27.38)
***Comorbidities [n (%)]***		
None	9 (90)	75 (95)
T2DM	0 (0)	2 (2.4)
Cardiomyopathy	0 (0)	1 (1.2)
Hypertension	1 (10)	1 (1.2)
Caspase-4 positive cells [mean (SEM)]	10.2 (1.3)	25.8 (1.2)

### Mice With Advanced Liver Fibrosis Treated With LPS Develop Multi-Organ Injury Associated With Activation of Hepatic Caspase-11 and GSDMD Cleavage

**FIGURE 2:**
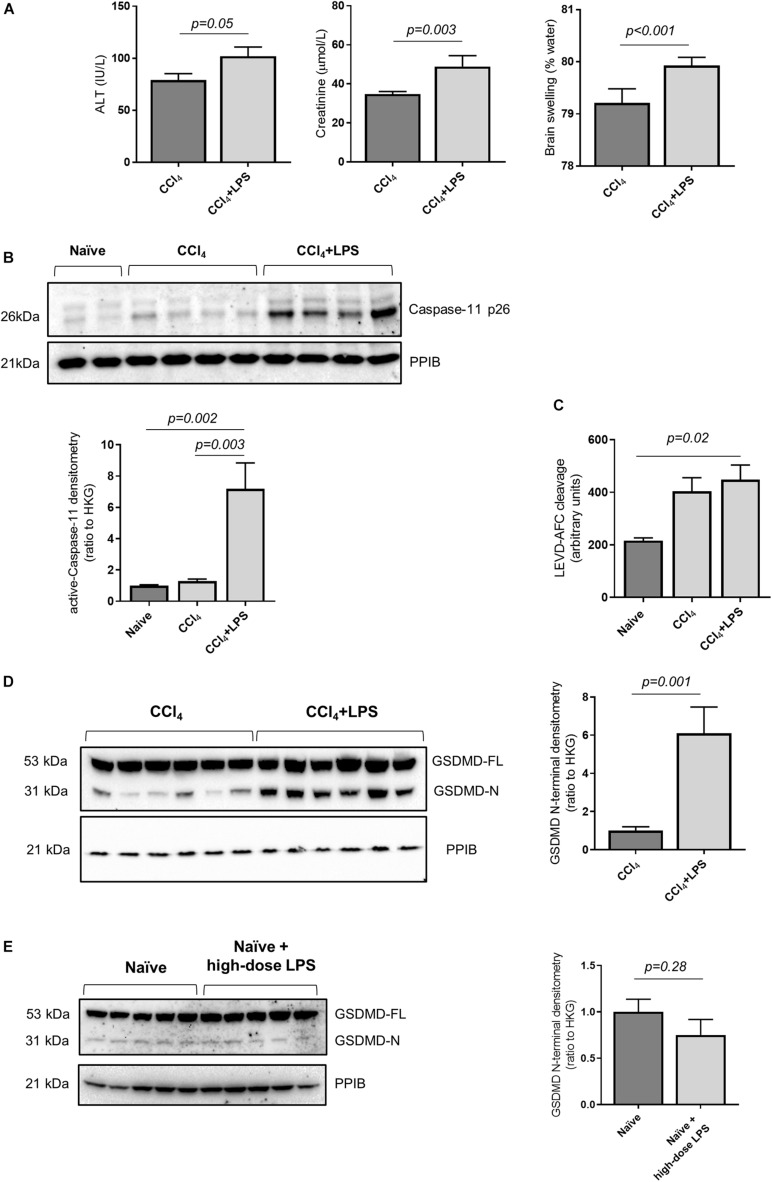
Mouse model of decompensated liver cirrhosis displays activity of Caspase-11 and GSDMD cleavage. **(A)** Mice were treated with CCl_4_ to develop advanced hepatic fibrosis. Following intraperitoneal injection of low-dose LPS (2 mg/kg), mice developed features of ACLF at 4 h (*n* = 7/group). The CCl_4_ + LPS group demonstrates elevated plasma ALT (left panel; Student’s *t*-test, *p* = 0.05), creatinine (center panel; Student’s *t*-test, *p* = 0.003) and brain swelling (right panel; Student’s *t*-test, *p* < 0.001). **(B)** Activation of caspase-11 was measured by relative abundance of active caspase-11 p26 compared to the housekeeping gene PPIB. CCl_4_ + LPS treated mice (*n* = 7; *n* = 6 showed in the image) showed higher levels of caspase-11 p26 than naïve mice (*n* = 5) and mice treated with CC_4_ alone (one-way ANOVA with Tukey *post hoc* test; *p* = 0.002 and *p* = 0.003, respectively). **(C)** The enzymatic cleavage of LEVD-AFC substrate in snap-frozen liver extracts showed a trend toward increase in the CCl_4_ group, and was significantly elevated in CCl_4_ + LPS-treated mice (*n* = 7) compared to naïve animals (*n* = 5) (one-way ANOVA with Tukey *post hoc* test *p* = 0.02). **(D)** Compared to animals treated with CCl_4_ alone, the CCl_4_ + LPS group shows higher expression of active GDSMD, measured as abundance of cleaved GSDMD N-terminal (Student’s *t*-test, *p* = 0.001). **(E)** There is no apparent increase in hepatic GSDMD activation in naïve mice or naïve mice treated with high-dose LPS (12 mg/kg) groups (Student’s *t*-test, *p* = 0.28).

In order to study the relationship between acute decompensation of cirrhosis with activation of the caspase-4/11 pathway, we utilized an established mouse model of liver fibrosis with multi-organ injury. Mice were treated with carbon tetrachloride (CCl_4_) over 10 weeks to establish advanced liver fibrosis (Supplementary Figure 1A), and subsequently injected intraperitoneally (i.p.) with LPS (2 mg/kg) to precipitate multi-organ injury. Mice treated with CCl_4_ + LPS developed features of ACLF, with exaggerated liver injury and extra-hepatic organ injury compared to CCl_4_ control (Figure 2A).

In accordance with the findings in humans, mice treated with CCl_4_ + LPS showed increased abundance of active caspase-11 in the liver compared to naïve mice and mice treated with CCl_4_ alone, as measured by abundance of the p26 fragment (Figure 2B). Further to that, the CCL_4_ + LPS group also displayed increased caspase-11 enzymatic activity, as measured by fluorometric assay on liver lysates (Figure 2C). consistent with activation and processing of caspase-11 following LPS exposure. Accordingly, circulating LPS levels, a marker of intestinal permeability and translocation of gut-derived bacterial products, showed a trend toward increased levels in the CCl_4_ and CCl_4_ + LPS groups compared to naïve (Supplementary Figure 1B). In contrast to caspase-11, no changes in the activity of caspase-1 were observed between naïve, CCl_4_ and CCl_4_ + LPS treated mouse liver samples (Supplementary Figure 1C). Taken together, these findings are consistent with increased activity, or ‘sensitization’, of the caspase-4/11 pathway in liver tissue in cirrhosis, predisposing to increased responsiveness to gut-derived bacterial products such as LPS.

Since caspase-11 acts upstream of GSDMD, we measured the abundance of active GSDMD by quantification of the GSDMD N-terminal. Mice treated with CCl_4_ + LPS showed increased levels of hepatic GSDMD N-terminal compared to the CCl_4_ control group (Figure 2D), which was accompanied by a significant increase in overall cell death in liver tissue, as assessed by TUNEL staining (Supplementary Figure 1D). In a separate experiment, naïve mice were treated with high-dose LPS (12 mg/kg) and showed no significant increase in hepatic GSDMD cleavage over baseline level in naïve mice (Figure 2E), thus suggesting that prior sensitization of the caspase-11 pathway is required to trigger hepatic GSDMD cleavage in response to LPS exposure.

Importantly, no increase in GSDMD cleavage was seen in the kidney or brain of the CCl_4_ + LPS group compared to CCl_4_ alone despite the increase in plasma creatinine and brain tissue water content noted in the CCl_4_ + LPS group (Supplementary Figure 2A). Representative liver and kidney H&E sections from naïve, CCl_4_, and CCl_4_ + LPS groups are presented in Supplementary Figure 2B.

### Endoplasmic Reticulum Stress Is Associated With the Upregulation in Caspase-11 Activity in Liver Tissue From Mice With Advanced Fibrosis

**FIGURE 3:**
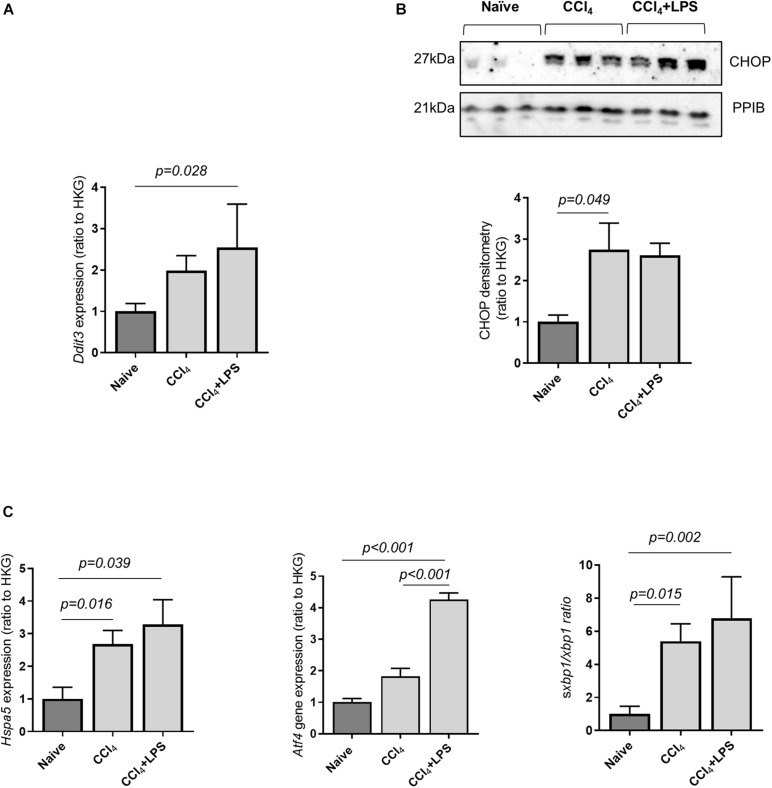
Cirrhotic mice display increased hepatic endoplasmic reticulum stress. The expression of key genes involved in ER stress were measured by qPCR and/or Western blot from snap-frozen liver extract in CCl_4_-treated (*n* = 7), and naïve (*n* = 5) mice. The housekeeping gene ppib was used for normalization across experiments. **(A)** Gene expression of the ER-stress marker *Ddit3*, coding for the protein CHOP, was increased in mice treated with CCl_4_ + LPS (Dunn’s test, *p* = 0.028). **(B)** Protein expression of mature CHOP was significantly increased in the CCl_4_-treated group, compared to control (Dunn’s test *p* = 0.049). **(C)** Gene expression of *Hspa5, Atf4, and ratio of spliced to unsplice xbp1* were significantly different across groups (one-way ANOVA: *Hspa5*, *p* = 0.039; *Atf4, p* < 0.001; s*Xbp1/Xbp1, p* = 0.002).

Prior work has demonstrated that hepatocyte ER stress is associated with liver fibrosis and cell death (Lebeaupin et al., 2015; Iracheta-Vellve et al., 2016). ER stress occurs as a consequence of disrupted intracellular homeostasis and accumulation of misfolded proteins, and has been associated with induction of caspase-11 activity through a direct interaction with the ER stress protein C/EBP homologous protein (CHOP) (Endo et al., 2006). Accordingly, CHOP expression was measured in liver tissue from naïve and CCl_4_-treated mice, and a significant upregulation of D*dit3* mRNA and CHOP protein expression was found in mice with advanced liver fibrosis compared to control (Figures 3A,B). ER stress was further confirmed by measurements of other markers genes: Hspa5, coding for the ER chaperone protein GRP78, Atf4, a stress-induced transcription factor, and spliced Xbp1, which were found to be elevated in the liver of CCl_4_-treated mice (Figure 3C). These results demonstrate an association between hepatic ER stress and upregulation of the caspase-11 pathway in CCl_4_-treated mice.

### Caspase-11 Deficient Mice Are Protected From Hepatic and Extra-Hepatic Organ Injury in ACLF

**FIGURE 4:**
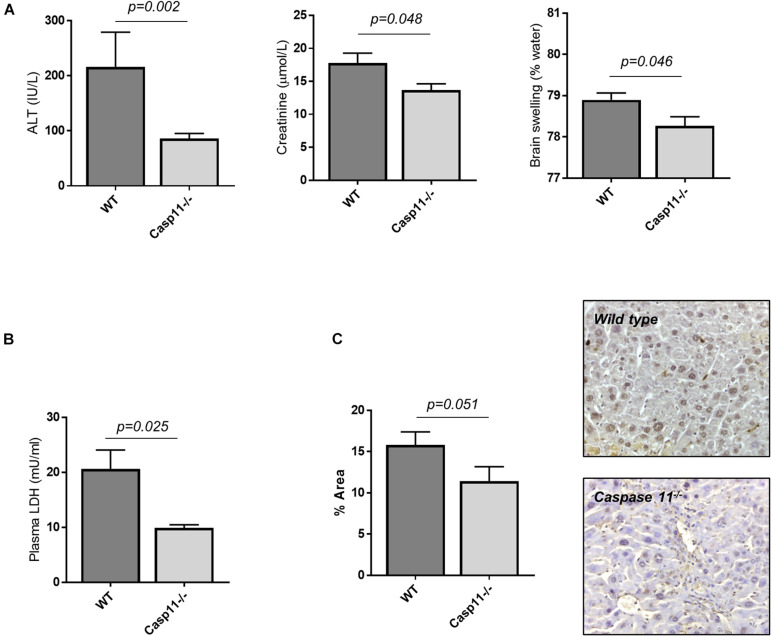
Caspase-11 deficiency protects from hepatic and extra-hepatic organ injury in ACLF. Wild type and c*asp11*^–/–^ type mice were treated with CCl_4_ (0.5ml/kg, 20 doses) to develop advanced hepatic fibrosis, and subsequently injected i.p., with LPS (4 mg/kg). **(A)** 4 h following LPS injection, *Casp11*^–/–^ mice (*n* = 6) displayed significantly lower ALT (Student’s *t*-test, *p* = 0.002), creatinine (Student’s *t*-test, *p* = 0.048), and brain tissue water content (Student’s *t*-test, *p* = 0.046) than wild type (*n* = 8). **(B)** Lower levels of cell death were also reflected in reduced levels of plasma LDH (Student’s *t*-test with Welch correction, *p* = 0.025). **(C)** Hepatocyte cell death was assessed by TUNEL assay and quantified by measuring positively stained areas. *Casp11*^–/–^ mice displayed lower levels of hepatocyte cell death than wild type (Student’s *t*-test, *p* = 0.051).

To explore the specific role of caspase-11 in the onset of multi-organ injury in response to LPS insult, we employed caspase-11 deficient mice (*Casp11*^–/–^ mice). Upon treatment with CCl_4_, *Casp11*^–/–^ mice developed a similar level of advanced fibrosis compared to wild-type (wt) mice (collagen proportionate area measurement: 5.3 ± 0.3 vs. 5.8 ± 0.5%, *p* = 0.36; result not shown), but were significantly protected from hepatic and extra-hepatic organ injury following i.p. injection of LPS (4 mg/kg) (Figure 4A). This was associated with a significant reduction in circulating LDH (Figure 4B) and in the number of TUNEL-positive cells on liver immunostaining (Figure 4C), consistent with a reduction in hepatocyte cell death. Absence of caspase-11 protein in the liver of *Casp11*^–/–^ mice following treatment with CCl_4_ and LPS was confirmed by western blotting (Supplementary Figure 3).

### Hepatocytes Undergo Pyroptosis in a Dose-Dependent Manner in Response to LPS, and Are “Sensitized” by Prior Low-Dose LPS Exposure and ER Stress

Prior data has shown that hepatocytes are the key cells undergoing apoptotic and non-apoptotic cell death in rodent models of ACLF (Adebayo et al., 2015). This observation was supported by TUNEL staining of liver tissue from CCl_4_ and CCl_4_ + LPS mice, which demonstrated primarily hepatocyte death following LPS injection, and a significant increase in overall cell death in the CCl_4_ + LPS group (Supplementary Figure 1D). Prior work has also demonstrated that hepatocytes can actively internalize LPS through carrier mechanisms, and that hepatocytes play a key role in the clearance of LPS during endotoxemia (Deng et al., 2013; Topchiy et al., 2016).

**FIGURE 5:**
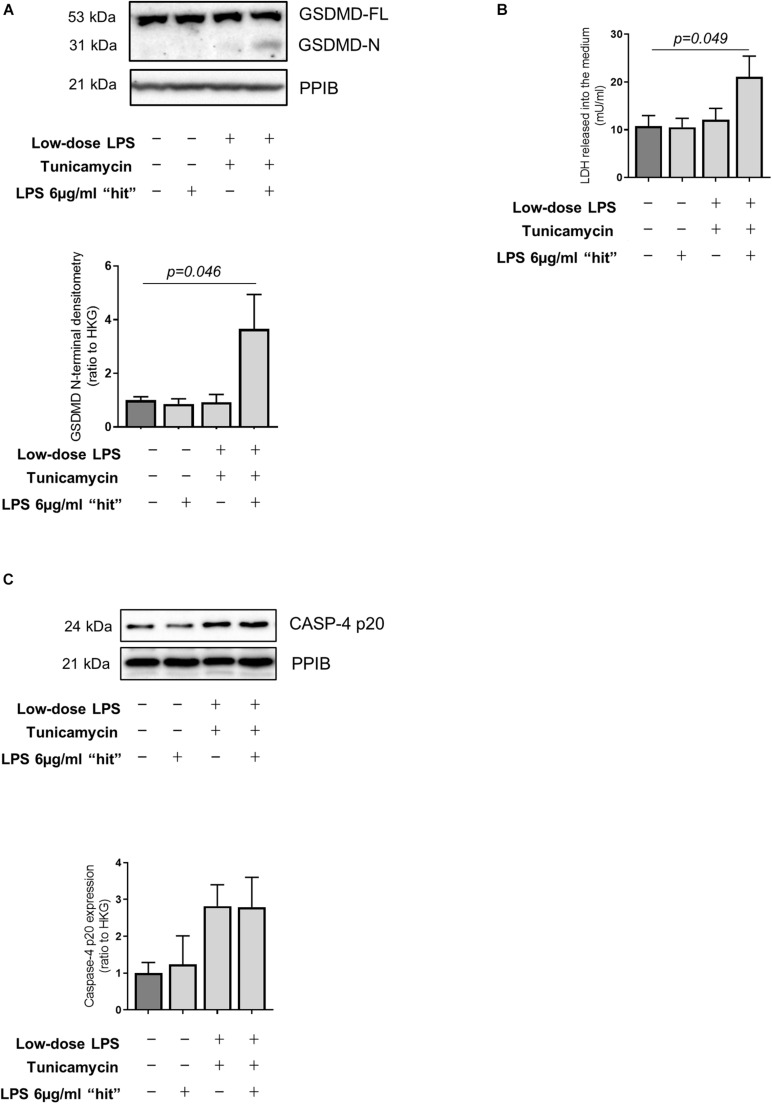
LPS-dependent pyroptosis in hepatocytes and sensitization by ER stress. **(A)** Primary human hepatocytes treated for 24 h with very low-dose LPS (100 ng/ml) and the ER-stress inducer tunicamycin (1 μM) prior to subsequent LPS ‘hit’ (6 μg/ml) show increased GSDMD cleavage compared to control (one-way ANOVA with Tukey *post hoc* test, *p* = 0.046, *n* = 3 experiments/group). **(B)** Susceptibility to induced pyroptosis was confirmed by increased release of LDH upon LPS “hit,” by hepatocytes treated with low-dose LPS (100 ng/ml) and tunicamycin (1 μM) (one-way ANOVA with Tukey *post hoc* test, *p* = 0.049, *n* = 3 experiments/group). **(C)** Primary human hepatocytes showed a trend toward increased active Caspase-4 upon tunicamycin treatment, as measured by the abundance of the p20 fragment.

Recent work has utilized a model of exposure of hepatocytes to low-dose LPS to mimic low-level gut-derived translocation of bacterial products in cirrhosis (Isaacs-Ten et al., 2020). Accordingly, we pre-treated primary human hepatocytes with low-dose LPS (100 ng/ml) alongside the ER stress inducer tunicamycin for 24 h to reflect the hepatocyte microenvironment in cirrhosis, prior to a subsequent LPS ‘hit’ (6 μg/ml). These experiments demonstrate that hepatocytes are resistant to pyroptosis in the baseline state but can be sensitized to pyroptosis in an environment of ER stress and low-dose LPS exposure, displaying increased abundance of GSDMD N-terminal (Figure 5A) and LDH release (Figure 5B). Treated hepatocytes also showed a trend toward increased activation of caspase-4, measured as abundance of the active (p20) fragment (Figure 5C), recapitulating the data collected *in vivo*.

## Discussion

In this study we demonstrate, for the first time, a role for LPS-sensing inflammatory caspases and the pyroptosis pathway in the progression to multi-organ injury in cirrhosis. The novel findings of this study are: (i) increased hepatic expression of caspase-4 is a feature of acutely decompensated cirrhosis and correlates with disease severity, (ii) hepatic expression of caspase-11 is also upregulated in a mouse model of advanced liver fibrosis, (iii) hepatic expression of the cleaved fragment of GSDMD, the effector protein of the pyroptosis pathway, is increased in mice with liver fibrosis and multi-organ injury, (iv) *Casp11*^–/–^ mice with advanced liver fibrosis are protected from excess hepatocyte death and multi-organ injury. These findings are of particular relevance, since cirrhosis is associated with progressive changes in the composition of the microbiome as well as translocation of bacterial-derived products (Bajaj, 2019). The work presented here provides a mechanistic link between bacterial dysbiosis and LPS translocation in cirrhosis, and LPS-mediated liver and multi-organ dysfunction (see graphical abstract). Moreover, these findings have translational importance through the potential utility of pyroptosis inhibitors as novel therapeutic strategies for acutely decompensated cirrhosis and ACLF.

Two groups have recently implicated activation of the pyroptosis pathways as a feature of fatty liver disease and alcohol-related liver disease. Xu et al. (2018) demonstrated increased expression of capases-1/4/5 and resulting GSDMD cleavage in patients with non-alcoholic steatohepatitis (NASH), with degree of cleavage correlating with hepatic inflammation. Further, they demonstrated protection from NASH in *Gsdmd*^–/–^ mice, and additionally used a hepatocyte-directed vector expressing GSDMD N-terminus to exacerbate hepatic injury. Khanova and colleagues also investigated the caspase-4/11-GSDMD pathway in alcoholic hepatitis and identified hepatic GSDMD cleavage as a feature of alcoholic hepatitis in mice and patients (Khanova et al., 2018). Additionally, they also used hepatocyte-specific over-expression of GSDMD N-terminus to exacerbate the alcoholic hepatitis phenotype, and demonstrated protection using *Casp1/Casp11* double knockout mice. Both these studies demonstrate a role for hepatocyte pyroptosis in steatohepatitis, although the mechanisms responsible for activation of caspase(s)-4/11 in liver disease remain ill-defined. The present study extends these previous observations and demonstrates a mechanistic link between translocation of gut-derived LPS and hepatocyte cell death in cirrhosis. Specifically, our study is the first to explore this pathway in advanced liver disease and to use mice with selective deficiency in caspase-11, thereby confirming a causal role for this pathway in multi-organ injury associated with decompensated cirrhosis. Importantly, these data are also consistent with human data, which demonstrate much higher levels of cell death in decompensated cirrhosis than in stable cirrhosis (Macdonald et al., 2018). Additionally, prior data supports a shift in mode of cell death, from apoptotic to non-apoptotic, with development of multi-organ failure in cirrhosis. Thus, although pyroptosis may be a feature of steatohepatitis, high levels of pyroptotic cell death are likely to play a pivotal role in the development of severe liver injury and multi-organ failure.

The data presented here also demonstrate that despite the occurrence of kidney injury and brain swelling in ACLF, there is no significant GSDMD cleavage in the kidney or brain in our mouse model of ACLF in response to LPS injection, suggesting that the injury observed in these organs is caused by mechanisms other than GSDMD-dependent pyroptosis. Indeed, the marked protection of the extra-hepatic organs in *Casp11*^–/–^ mice suggests that the primary event is hepatic pyroptosis subsequently leading to extra-hepatic organ injury, rather than direct LPS-induced injury in these extra-hepatic organs. The recently described roles of extracellular vesicles in inter organ communication and the correlation between liver injury and increased levels of extracellular vesicle release from hepatocytes (Malhi, 2019), warrant investigation into whether they might be conduit of the signal leading to multi-organ failure in response to liver injury in cirrhosis.

Currently, there are no specific therapies for acutely decompensated cirrhosis and ACLF, and previous attempts to modify cell death pathways in this group, through pan-caspase inhibition, have been unsuccessful (Mehta et al., 2018). The data presented here suggest that a more targeted approach to inhibit inflammatory caspases or downstream pyroptosis pathways would be preferable. Recent work from Hu et al. demonstrates that disulfiram, a licensed drug with an excellent safety record, is an inhibitor of pyroptosis (Hu et al., 2020). Consequently, it is clear that disulfiram is an attractive candidate for repurposing as a potential therapeutic for cirrhosis and ACLF, and clinical trials are warranted.

The limitations of this work are the skewed distribution of patients between the AD and SC groups, which is however justified by the limited variability among the SC patients, and that we cannot exclude a role for pyroptosis in hepatic phagocytic cells, such as infiltrating bone-marrow derived monocytes or Kupffer cells, in the development of ACLF. The immunostaining data, in mice and humans, presented here demonstrate primarily hepatocyte staining in liver tissue samples, although the involvement of monocyte-macrophage cells cannot be absolutely excluded histologically. The data presented here are also consistent with the findings from Khanova et al. (2018) and Xu et al. (2018) in previous studies who noted primarily hepatocyte pyroptosis in disease models, as well as clinical data from the CANONIC cohort study which demonstrate that circulating markers of cell death in ACLF are correlated with markers of hepatic injury (bilirubin, alanine aminotransferase) rather than markers of extra-hepatic organ dysfunction (Moreau et al., 2013; Khanova et al., 2018; Xu et al., 2018). Fundamentally, these findings build upon an existing body of work supporting a specific role of hepatocytes in handling circulating LPS, as distinct from the innate immune compartment which plays a primary role in responding to microorganisms and DAMPs (Mimura et al., 1995; Deng et al., 2013; Topchiy et al., 2016). Our data further suggests that the handling of circulating LPS in cirrhosis is aberrant due to sensitization of the hepatocyte caspase-4/11 pathway.

## Conclusion

In this study we demonstrate for the first time upregulation of inflammatory caspase(s)-4/11 and increased hepatocyte pyroptosis in acutely decompensated cirrhosis, and a causal link between translocation of gut-derived LPS and liver- and multi-organ injury in mouse models of liver fibrosis. This work highlights pyroptosis as a potential novel target for therapy in patients with acutely decompensated cirrhosis and ACLF.

## Data Availability Statement

The original contributions presented in the study are included in the article/Supplementary Material, further inquiries can be directed to the corresponding author/s

## Ethics Statement

The studies involving human participants were reviewed and approved by Institute of Liver and Biliary Sciences, Institutional Review Board (IRB) – 02/11/2019. Written informed consent for participation was not required for this study in accordance with the National Legislation and the institutional requirements. The animal study was reviewed and approved by UCL Animal Welfare and Ethical Review Body (AWERB).

## Author Contributions

US and NB contributed to experimental design, data collection and analysis, and drafted the article. EW, SB, CB, AbH, AS, ViP, VaP, AnH, TL, and AN contributed to data collection and drafted the article. RW, SC, BP, RM, and RJ contributed to experimental design and drafted the article. GM supervised the study, contributed to experimental design, data collection and analysis, and drafted the article. All authors contributed to the article and approved the submitted version.

## Conflict of Interest

RJ has research collaborations with Takeda and Yaqrit, consults for Mallinckrodt and Yaqrit and has received speaking fees from Grifols, and founder of Yaqrit Limited and Thoeris GmBh, which is developing UCL inventions for treatment of patients with cirrhosis. The remaining authors declare that the research was conducted in the absence of any commercial or financial relationships that could be construed as a potential conflict of interest.

## Abbreviations

ACLF: acute-on-chronic liver failure
AD: acutely decompensated cirrhosis
ALT: alanine aminotransferase
ATF4: activating transcription factor 4
CCl_4_: carbon tetrachloride
CHOP: C/EBP homologous protein
CPA: collagen proportionate area
DAMP: damage-associated molecular pattern
DC: decompensated cirrhosis
DDIT3: DNA damage-inducible transcript 3 (gene coding for CHOP protein)
ER: endoplasmic reticulum
GAPDH: glyceraldehyde 3-phosphate dehydrogenase
GSDMD: gasdermin-D
HC: healthy control
HE: hepatic encephalopathy
HSPA5: heat shock protein family A (Hsp70) member 5 (gene coding for BiP)
LDH: lactate dehydrogenase
LPS: lipopolysaccharide/bacterial endotoxin
MELD: model for end-stage liver disease
PAMP: pathogen-associated molecular pattern
PPIB: peptidylprolyl isomerase B
SC: stable cirrhosis
TUNEL: terminal deoxynucleotidyl transferase dUTP nick end labeling
XBP1: X-box binding protein 1.

## References

[B1] Adebayo D. Morabito V. Andreola F. Pieri G. Luong T. V. Dhillon A. (2015). Mechanism of cell death in acute-on-chronic liver failure: a clinico-pathologic-biomarker study. *Liver Int.* 35 2564–2574. 10.1111/liv.12850 25879577

[B2] Bajaj J. S. (2019). Altered Microbiota in Cirrhosis and Its Relationship to the Development of Infection. *Clin. Liver Dis.* 14 107–111. 10.1002/cld.827 31632660 PMC6784803

[B3] Carl D. E. Ghosh S. S. Gehr T. W. Abbate A. Toldo S. Sanyal A. J. (2016). A model of acute kidney injury in mice with cirrhosis and infection. *Liver Int.* 36 865–873. 10.1111/liv.13023 26583566

[B4] Clària J. Arroyo V. Moreau R. (2016). The Acute-on-Chronic Liver Failure Syndrome, or When the Innate Immune System Goes Astray. *J. Immunol.* 197 3755–3761. 10.4049/jimmunol.1600818 27815438

[B5] Deng M. Scott M. J. Loughran P. Gibson G. Sodhi C. Watkins S. (2013). Lipopolysaccharide clearance, bacterial clearance, and systemic inflammatory responses are regulated by cell type-specific functions of TLR4 during sepsis. *J. Immunol.* 190 5152–5160. 10.4049/jimmunol.1300496 23562812 PMC3644895

[B6] Ding J. Wang K. Liu W. She Y. Sun Q. Shi J. (2016). Pore-forming activity and structural autoinhibition of the gasdermin family. *Nature* 535 111–116. 10.1038/nature18590 27281216

[B7] Endo M. Mori M. Akira S. Gotoh T. (2006). C/EBP Homologous Protein (CHOP) Is Crucial for the Induction of Caspase-11 and the Pathogenesis of Lipopolysaccharide-Induced Inflammation. *J. Immunol.* 176 6245–6253. 10.4049/jimmunol.176.10.6245 16670335

[B8] Gandoura S. Weiss E. Rautou P. E. Fasseu M. Gustot T. Lemoine F. (2013). Gene- and exon-expression profiling reveals an extensive LPS-induced response in immune cells in patients with cirrhosis. *J. Hepatol.* 58 936–948. 10.1016/j.jhep.2012.12.025 23321315

[B9] Hall A. R. Tsochatzis E. Morris R. Burroughs A. K. Dhillon A. P. (2013). Sample size requirement for digital image analysis of collagen proportionate area in cirrhotic livers. *Histopathology* 62 421–430. 10.1111/his.12010 23134419

[B10] Hu J. J. Liu X. Zhao J. Xia S. Zhang Z. Zhang Y. (2020). FDA-approved disulfiram inhibits pyroptosis by blocking gasdermin D pore formation. *Nat. Immunol.* 21 736–745. 10.1038/s41590-020-0669-6 32367036 PMC7316630

[B11] Iracheta-Vellve A. Petrasek J. Gyongyosi B. Satishchandran A. Lowe P. Kodys K. (2016). Endoplasmic reticulum stress-induced hepatocellular death pathways mediate liver injury and fibrosis via Stimulator of Interferon Genes. *J. Biol. Chem.* 291 26794–26805. 10.1074/jbc.m116.736991 27810900 PMC5207187

[B12] Isaacs-Ten A. Echeandia M. Moreno-Gonzalez M. Brion A. Goldson A. Philo M. (2020). Intestinal microbiome-macrophage crosstalk contributes to cholestatic liver disease by promoting intestinal permeability. *Hepatology* 72 2090–2108. 10.1002/hep.31228 32168395 PMC7839474

[B13] Khanova E. Wu R. Wang W. Yan R. Chen Y. French S. W. (2018). Pyroptosis by caspase11/4-gasdermin-D pathway in alcoholic hepatitis in mice and patients. *Hepatology* 67 1737–1753. 10.1002/hep.29645 29108122 PMC5906140

[B14] Lebeaupin C. Proics E. de Bieville C. H. Rousseau D. Bonnafous S. Patouraux S. (2015). ER stress induces NLRP3 inflammasome activation and hepatocyte death. *Cell Death Dis.* 6:e1879. 10.1038/cddis.2015.248 26355342 PMC4650444

[B15] Macdonald S. Andreola F. Bachtiger P. Amoros A. Pavesi M. Mookerjee R. (2018). Cell death markers in patients with cirrhosis and acute decompensation. *Hepatology* 67 989–1002. 10.1002/hep.29581 29023872

[B16] Malhi H. (2019). Emerging role of extracellular vesicles in liver diseases. *Am. J. Physiol. Gastrointest. Liver Physiol.* 317 G739–G749. 10.1152/ajpgi.00183.2019 31545919 PMC6879890

[B17] Markwick L. J. L. Riva A. Ryan J. M. Cooksley H. Palma E. Tranah T. H. (2015). Blockade of PD1 and TIM3 restores innate and adaptive immunity in patients with acute alcoholic hepatitis. *Gastroenterology* 148 590–602. 10.1053/j.gastro.2014.11.041 25479137

[B18] Marmarou A. Tanaka K. Shulman K. (1982). An improved gravimetric measure of cerebral edema. *J. Neurosurg.* 56 246–253. 10.3171/jns.1982.56.2.0246 7054433

[B19] Mehta G. Rousell S. Burgess G. Morris M. Wright G. McPherson S. (2018). A Placebo-Controlled, Multicenter, Double-Blind, Phase 2 Randomized Trial of the Pan-Caspase Inhibitor Emricasan in Patients with Acutely Decompensated Cirrhosis. *J. Clin. Exp. Hepatol.* 8 224–234. 10.1016/j.jceh.2017.11.006 30302038 PMC6175779

[B20] Merion R. M. Wolfe R. A. Dykstra D. M. Leichtman A. B. Gillespie B. Held P. J. (2003). Longitudinal assessment of mortality risk among candidates for liver transplantation. *Liver Transpl.* 9 12–18. 10.1053/jlts.2003.50009 12514767

[B21] Michelena J. Altamirano J. Abraldes J. G. Affò S. Morales-Ibanez O. Sancho-Bru P. (2015). Systemic inflammatory response and serum lipopolysaccharide levels predict multiple organ failure and death in alcoholic hepatitis. *Hepatology* 62 762–772. 10.1002/hep.27779 25761863 PMC4549175

[B22] Mimura Y. Sakisaka S. Harada M. Sata M. Tanikawa K. (1995). Role of hepatocytes in direct clearance of lipopolysaccharide in rats. *Gastroenterology* 109 1969–1976. 10.1016/0016-5085(95)90765-3 7498663

[B23] Mokdad A. A. Lopez A. D. Shahraz S. Lozano R. Mokdad A. H. Stanaway J. (2014). Liver cirrhosis mortality in 187 countries between 1980 and 2010: a systematic analysis. *BMC Med.* 12:145. 10.1186/s12916-014-0145-y 25242656 PMC4169640

[B24] Moreau R. Jalan R. Gines P. Pavesi M. Angeli P. Cordoba J. (2013). Acute-on-chronic liver failure is a distinct syndrome that develops in patients with acute decompensation of cirrhosis. *Gastroenterology* 144 1426–1437. 10.1053/j.gastro.2013.02.042 23474284

[B25] Tazi K. A. Bièche I. Paradis V. Guichard C. Laurendeau I. Dargère D. (2007). In vivo altered unfolded protein response and apoptosis in livers from lipopolysaccharide-challenged cirrhotic rats. *J. Hepatol.* 46 1075–1088. 10.1016/j.jhep.2007.01.034 17399843

[B26] Topchiy E. Cirstea M. Kong H. J. Boyd J. H. Wang Y. Russell J. A. (2016). Lipopolysaccharide is cleared from the circulation by hepatocytes via the low density lipoprotein receptor. *PLoS One* 11:e0155030. 10.1371/journal.pone.0155030 27171436 PMC4865154

[B27] Wang S. Miura M. Jung Y. K. Zhu H. Li E. Yuan J. (1998). Murine caspase-11, an ICE-interacting protease, is essential for the activation of ICE. *Cell* 92 501–509. 10.1016/s0092-8674(00)80943-5 9491891

[B28] Xu B. Jiang M. Chu Y. Wang W. Chen D. Li X. (2018). Gasdermin D plays a key role as a pyroptosis executor of non-alcoholic steatohepatitis in humans and mice. *J. Hepatol.* 68 773–782. 10.1016/j.jhep.2017.11.040 29273476

