# Supplementary material for: The Lipopolysaccharide-Sensing Caspase(s)-4/11 Are Activated in Cirrhosis and Are Causally Associated With Progression to Multi-Organ Injury
Source: Front Cell Dev Biol. 2021 Jul 15;9:668459. doi: 10.3389/fcell.2021.668459 (PMC8320658; doi:10.3389/fcell.2021.668459)
Supplement: Supplementary file 1 [file Data_Sheet_1.docx]

Supplementary Material

| **Species** | **Host** | **Target** | **Manufacturer** | **Cat. #** |
| --- | --- | --- | --- | --- |
| Mouse | Rabbit | GSDMD | Abbexa | ABX340202 |
| Mouse | Rabbit | Caspase-11 | Abcam | ab180673 |
| Mouse | Mouse | CHOP | Abcam | ab11419 |
| Mouse | Rabbit | PPIB | Biorbyt | orb252737 |
| Human^*^ | Rabbit | Caspase-4 | AMS bio | E-AB-53537-60 |
| Human**^‡^** | Rabbit | Caspase-4 | Biotechne | NBP1-77208 |

**Supplemental table 1a: List of primary antibodies (^*^anti-caspase-4 antibody used for IHC, ^‡^anti-caspase-4 antibody used for western blots)**

| **Gene name** | **Symbol** | **Forward** | **Reverse** |
| --- | --- | --- | --- |
| Peptidylprolyl isomerase B (housekeeping gene) | *Ppib* | AGGACTTCATGATCCAGGGTGGAGA | TGGTGTCTTTGCCTGCATTGGC |
| DNA Damage Inducible Transcript 3 | *Ddit3* | CATACACCACCACACCTGAAAG | CCGTTTCCTAGTTCTTCCTTGC |
| Activating Transcription Factor 4 | *Atf4* | ACCTATAAAGGCTTGCGGCCACC | GCAACACTGCTGCTGGATTTCGT |
| Heavy chain binding protein | *HSpa5* | TTGGAGGTGGGCAAACCAAGACA | TTGGTTGCTTGTCGCTGGGC |
| XBP1 variant 1 | *Xbp1* | AGAAAGCCCGGATGAGCGAGC | CCTGCTGCAGAGGTGCACATAGT |
| XBP1 spliced | *sXbp1* | GGTCTGCTGAGTCCGCAGCAGG | ACTAGCAGACTCTGGGGAAGGACAT |

**Supplemental table 1b: Mouse primers used for gene expression analysis**

| **Item** | **Supplier** | **Cat. #** |
| --- | --- | --- |
| Lipopolysaccharides from *Klebsiella pneumoniae* | Merck | L4268 |
| In Situ Cell Death Detection Kit | Merck | 11684817910 |
| LDH-Glo Cytotoxicity Assay | Promega | J2380 |
| LAL Chromogenic Endotoxin Quantitation Kit | ThermoFisher Scientific | 88282 |
| Tunicamycin from Streptomyces sp | Merck | T7765 |
| RIPA Lysis Buffer, 10X | Merck | 20-188 |
| ECL Western Blotting Substrates | BioRad | 1705060 |
| TRI-reagent | Merck | T9424 |
| AffinityScript QPCR cDNA Synthesis Kit | Agilent | 600559 |
| Caspase-1 Assay Kit | Abcam | ab39412 |
| Caspase-11 Assay Kit | Abcam | ab65658) |

**Supplemental table 1c: List of reagents**


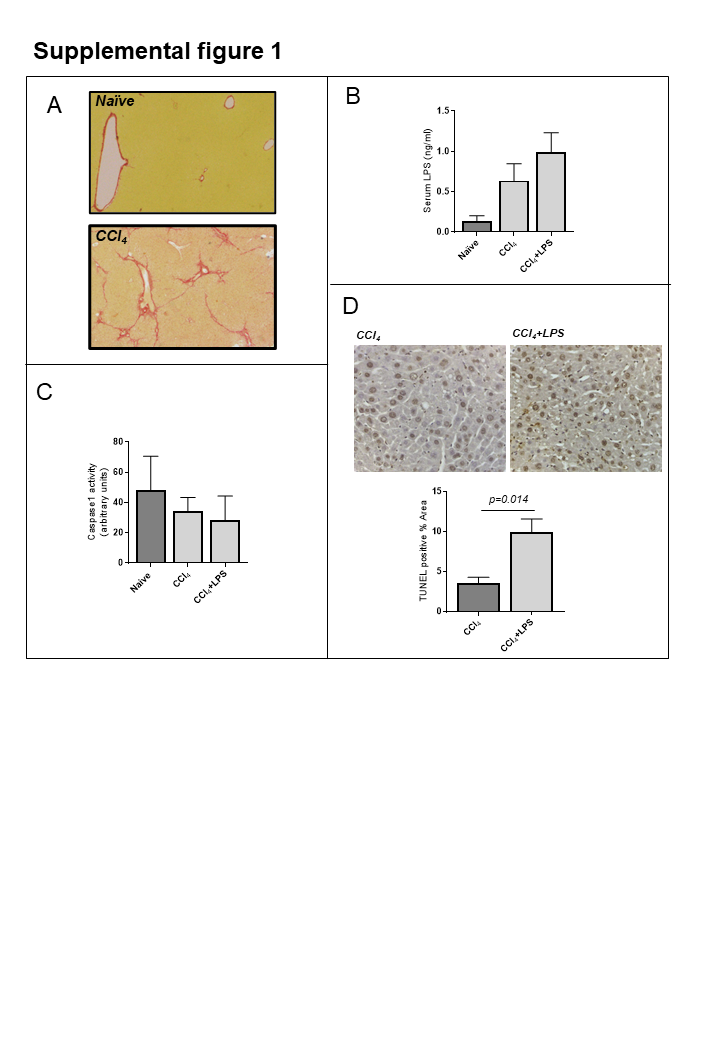


**Supplemental figure 1.** (A) Mice treated with 20 doses of CCl_4_ developed advanced fibrosis compared to control (representative Sirius red stain). (B) Circulating LPS levels were measured by chromogenic assay, and showed a trend towards increased levels in the CCl_4_ and CCl_4_+LPS (2mg/kg) groups compared to naïve. (C) The enzymatic activity of caspase-1 in snap-frozen liver extracts was unchanged in naïve mice (n=5) compared to CCl_4_-treated mice (n=7) compared to naïve animals (n=5). (D) Mice treated with CCl_4_+LPS (2mg/kg) demonstrate increased hepatocyte cell death as assessed by TUNEL assay compared to mice treated with CCl_4_ alone (Mann Whitney test, p=0.014).


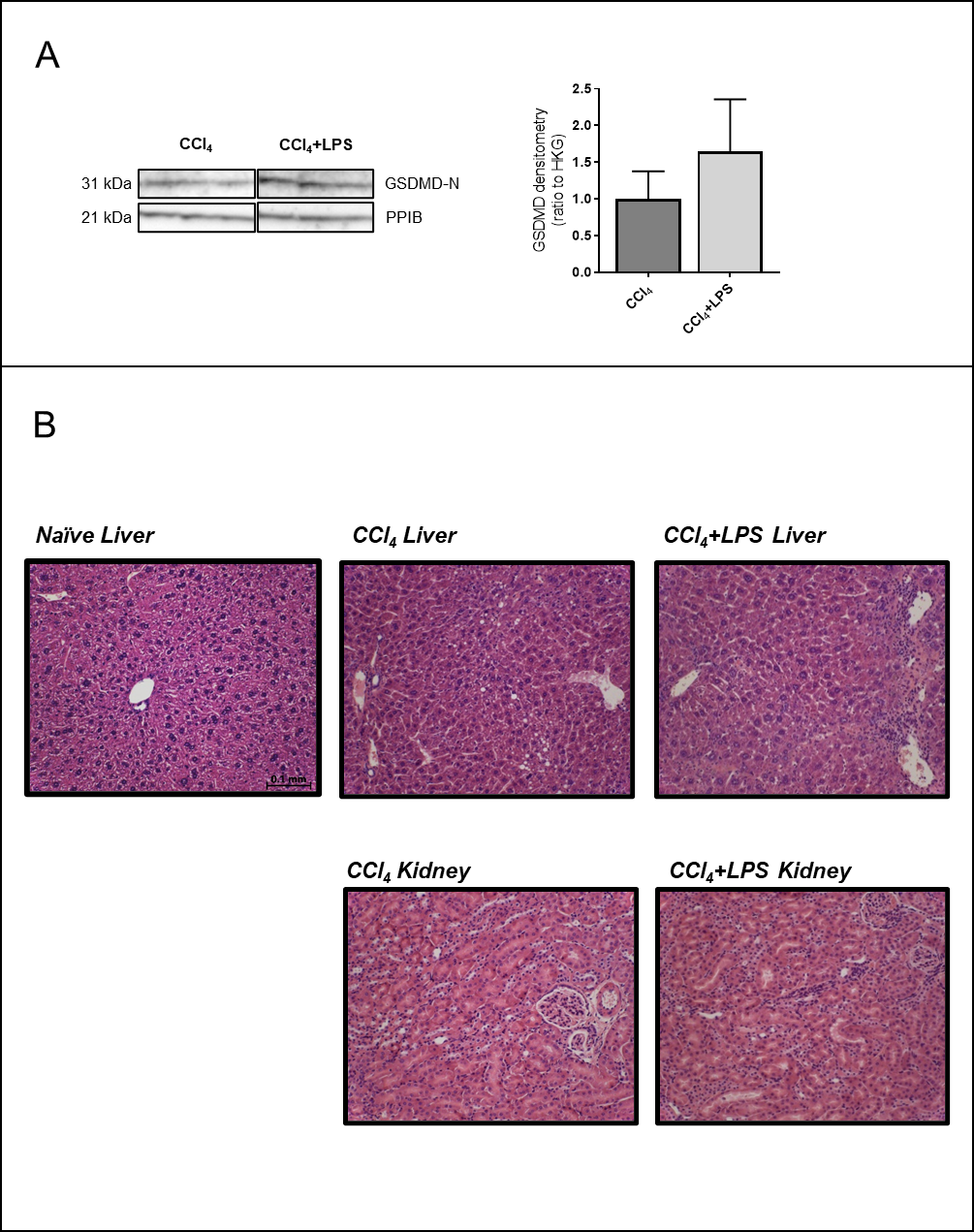


**Supplemental figure 2.** (A) Mice treated with CCl_4_+LPS do not show increased processing of GSDMD in the kidneys compared to animals treated with CCl_4_ alone, as measured by the abundance of GSDMD N-terminal relative to the housekeeping gene PPIB. (B) H&E staining of mouse liver and kidney from CCl_4_ and CCl_4_+LPS groups


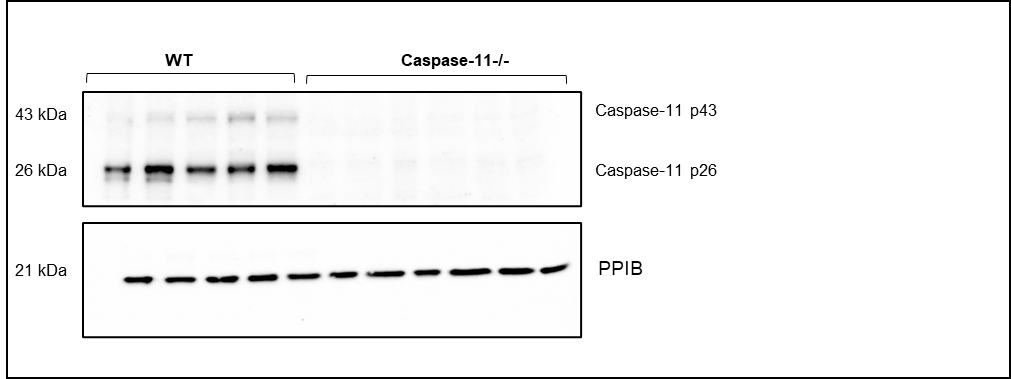


**Supplemental figure 3.** Abundance of caspase-11 protein was measured by Western blot in snap-frozen liver from *wt* and *Casp-11^-/-^* mice treated with CCl_4_ and LPS (4mg/kg).
